# Contrast-enhanced MR imaging of pulmonary arteries: new imaging strategies using different contrast agents

**DOI:** 10.1186/1532-429X-11-S1-P63

**Published:** 2009-01-28

**Authors:** Marcus R Makowski, Andrea J Wiethoff, Vicky Parish, Aaron Bell, Rene M Botnar, Christian Jansen, Sergio Uribe, Martin Rohrer, Reza Razavi, Tobias Schaeffter, Gerald F Greil

**Affiliations:** 1 Imaging Sciences Division, King's College London, St Thomas' Hospital, London, UK; 2 NIHR Biomedical Research Centre at Guy's & St Thomas' Hospital and King's College London, London, UK; 3 European Business Unit Diagnostic Imaging, Bayer Schering Pharma AG, Berlin, Germany

**Keywords:** Inversion Recovery, Breath Hold, Pulmonary Vasculature, Contrast Enhance Magnetic Resonance Angiography, Respiratory Navigator

## Objective

First pass breath hold non ECG triggered 3D Gd-DTPA contrast enhanced magnetic resonance angiography (CEMRA) is commonly used for morphologic assessment of the pulmonary arteries. However, image resolution is limited due to time constraints and vascular borders are blurred due to vascular motion and insufficient breath holds.

## Background

Pulmonary vascular imaging using a respiratory gated and ECG triggered 3D contrast enhanced IR prepulse sequence in combination with Gadofosveset (mean intravascular t1/2α = 0.48 ± 0.11 h) and 32 channel coil technology yields significant higher pulmonary vascular detail compared to breath hold 3D CEMRA.

## Methods

In eight subjects (29 ± 6 yrs) with normal pulmonary vasculature CEMRA was performed on a 1.5 T clinical scanner (Philips Medical Systems). Patients were investigated twice within 7 days using Gd-DTPA (day 1, 0.10–0.17 mmol/kg) and Gadofosveset (day 2, 0.03 mmol/kg). 3D CEMRA as well as a respiratory navigator gated and ECG triggered 3D steady-state free precession (SSFP) sequence with a T2 prepulse were used on both days. An inversion recovery (IR) prepulse to suppress surrounding tissue signal was applied with Gadofosveset. Results were quantitatively and qualitatively (image quality: 1 = non diagnostic, 2 = diagnostic, 3 = good 4 = excellent) compared.

## Results

**Figure 1:**
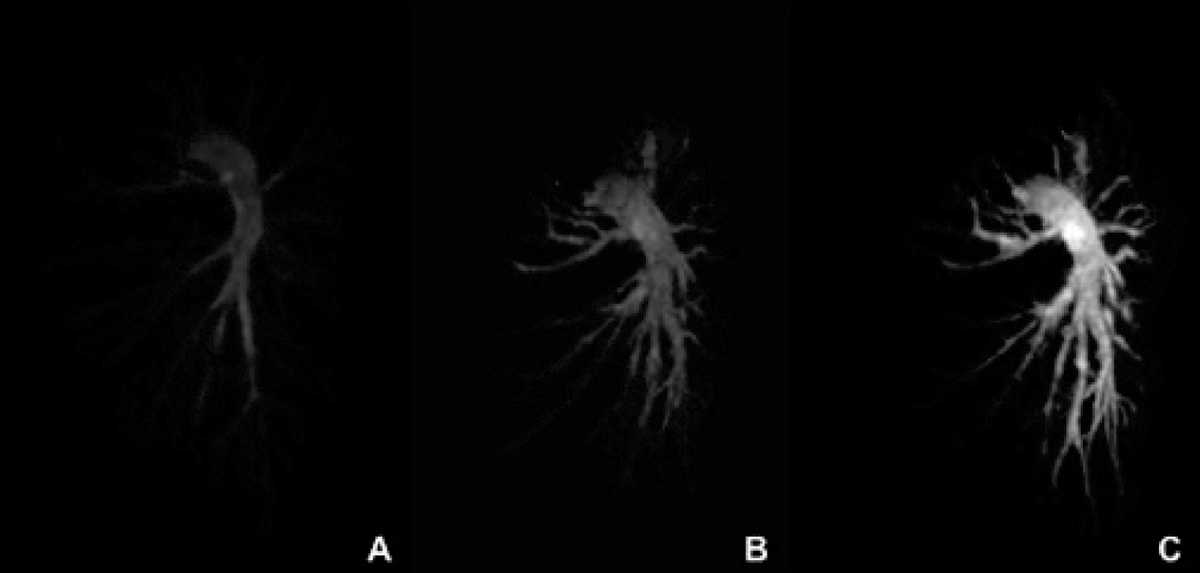
Figure 1

Best results were achieved for the high resolution navigator gated and ECG triggered 3D IR SSFP sequence using Gadofosveset (Figure 1C) regarding CNR, vessel length, vessel wall sharpness and image quality (Table 1). Gadofosveset did not improve image quality in the 3D SSFP technique without IR (Figure 1B) compared to Gd-DTPA and 3D first pass CEMRA (Figure 1A).

**Table 1:** Comparison of contrast agents and imaging sequences

Contrast Agent	Sequence	Vessel length (cm)	Vessel sharpness (%)	Contrast to Noise Ratio (CNR)	Image quality (mean ± SD)	Isotropic spatial resolution (mm^3^)
**Gd-DTPA**	CEMRA	14 ± 4‡	33 ± 6‡	89 ± 37‡	2.1 ± 1.2‡	1.77
	SSFP	17 ± 2*	41 ± 4*	134 ± 22*	3.2 ± 1.1*	1.49
**Gadofosveset**	CEMRA	13 ± 4‡	34 ± 7‡	99 ± 39‡	2.3 ± 1.1‡	1.77
	SSFP	17 ± 2*	40 ± 4*	136 ± 17*	3.1 ± 1.5*	1.49
	SSFP+IR	19 ± 3^†^	47 ± 5^†^	153 ± 24^†^	3.6 ± 1.3^†^	1.49

*p < 0.05 vs CEMRA, ^†^p < 0.05 vs SSFP (same CA); no significant differences between corresponding sequences (Gd-DTPA vs Gadofosveset)

## Conclusion

Pulmonary vascular imaging using a navigator gated and ECG triggered 3D IR SSFP sequence with Gadofosveset and 32 channel coil technology yielded significantly higher morphologic detail compared to breath hold CEMRA and 3D SSFP without IR. This technique has the potential to improve diagnostic imaging of the pulmonary vasculature.

